# Functional annotation of novel lineage-specific genes using co-expression and promoter analysis

**DOI:** 10.1186/1471-2164-11-161

**Published:** 2010-03-09

**Authors:** Charu G Kumar, Robin E Everts, Juan J Loor, Harris A Lewin

**Affiliations:** 1 Department of Animal Sciences, University of Illinois at Urbana-Champaign, 210 Edward R Madigan Laboratory, 1201 W Gregory Dr, Urbana, IL 61801, USA; 2 Institute for Genomic Biology, University of Illinois at Urbana-Champaign, 1206 West Gregory Drive, Room 1608, Urbana, IL 61801, USA; 3 Current address: SEQUENOM, Inc, 3595 John Hopkins Court, San Diego, CA 92121, USA

## Abstract

**Background:**

The diversity of placental architectures within and among mammalian orders is believed to be the result of adaptive evolution. Although, the genetic basis for these differences is unknown, some may arise from rapidly diverging and lineage-specific genes. Previously, we identified 91 novel lineage-specific transcripts (LSTs) from a cow term-placenta cDNA library, which are excellent candidates for adaptive placental functions acquired by the ruminant lineage. The aim of the present study was to infer functions of previously uncharacterized lineage-specific genes (LSGs) using co-expression, promoter, pathway and network analysis.

**Results:**

Clusters of co-expressed genes preferentially expressed in liver, placenta and thymus were found using 49 previously uncharacterized LSTs as seeds. Over-represented composite transcription factor binding sites (TFBS) in promoters of clustered LSGs and known genes were then identified computationally. Functions were inferred for nine previously uncharacterized LSGs using co-expression analysis and pathway analysis tools. Our results predict that these LSGs may function in cell signaling, glycerophospholipid/fatty acid metabolism, protein trafficking, regulatory processes in the nucleus, and processes that initiate parturition and immune system development.

**Conclusions:**

The placenta is a rich source of lineage-specific genes that function in the adaptive evolution of placental architecture and functions. We have shown that co-expression, promoter, and gene network analyses are useful methods to infer functions of LSGs with heretofore unknown functions. Our results indicate that many LSGs are involved in cellular recognition and developmental processes. Furthermore, they provide guidance for experimental approaches to validate the functions of LSGs and to study their evolution.

## Background

Placentae exhibit remarkable variation in tissue structure and morphology within and between mammalian clades, and even within a single mammalian order [1]. The diversity of placental architectures is thought to be the result of adaptive evolution arising from rapidly diverging and novel genes [2-4]. A greater understanding of the functional roles that these genes play would provide insights into the molecular basis for the unique phenotypic and metabolic adaptations among closely related mammalian species. Toward that end, we previously identified and bioinformatically characterized novel transcripts in cattle using placenta as a source tissue [2]. These transcripts are lineage-specific (LSTs), and the genes that encode them have no detectable homology to genes outside of that lineage (LSGs). Functional elucidation of LSGs remains a daunting task and only a few have been characterized beyond their expression patterns [5-10]. A complementary approach that would direct the genetic and biochemical characterization of LSGs and their products is functional inference using co-expression [11] and promoter analysis [12].

Gene expression is regulated by a complex interaction of transcription factors (TFs) and their binding sites (TFBS) on the gene promoter. Co-expression analysis is based upon the assumption that a high degree of similarity in gene expression profiles correlates with relatedness of their functions [11]. Genes that are highly co-expressed are often regulated by common transcription factor(s), forming sub-networks of genes with a common function [12]. As a general rule, co-regulated genes share a specific arrangement of TFBSs on their promoters. The TFBSs are often located in a specific order relative to the transcription start site (TSS) as well as in a particular orientation with respect to the promoter [13]. For example, Kindy et al. [14] showed that both strands of the *c-myc *gene are transcribed in an overlapping fashion and that transcription of the coding and non-coding strands is regulated independently. Yu and coauthors [15] showed a strong correlation between inter-TFBS distances and their orientation with respect to each other, demonstrating that a combination of TFs rather than an individual TF is the functional unit in tissue-specific gene regulation. Others have shown that the inter-TFBS distance between functionally over-represented TFBS pairs can vary significantly from 10 to 200 bp, although it may be greater than 200 bp in some cases [16-18]. These findings provide insights into factors governing the interactions between specific TFs and document TF pairs that are predicted to act synergistically in a tissue-specific manner [19] or at specific stages of development [20].

In a previous work we identified 91 cattle- and cetartiodactyl-specific novel transcripts that included coding sequences and noncoding RNAs (ncRNAs) [2]. In the present work, we have inferred functions of a subset of these LSTs using co-expression analysis. In addition, we identified over-represented TFBSs and their composites in the promoters of co-expressed genes and searched existing databases and the literature for pathways and functions in which these TFs may play a synergistic role in a specific tissue or developmental stage. Using these functional inferences, we predicted sub-networks of genes that may be co-regulated with the LSGs. Our results predict that subsets of these LSGs function in glycerophospholipid/fatty acid metabolism and protein trafficking in liver and near-term placenta, and in processes involving the initiation of parturition and immune system development.

## Results

### Identification of tissue-specific and time-series clusters

**Figure 1:**
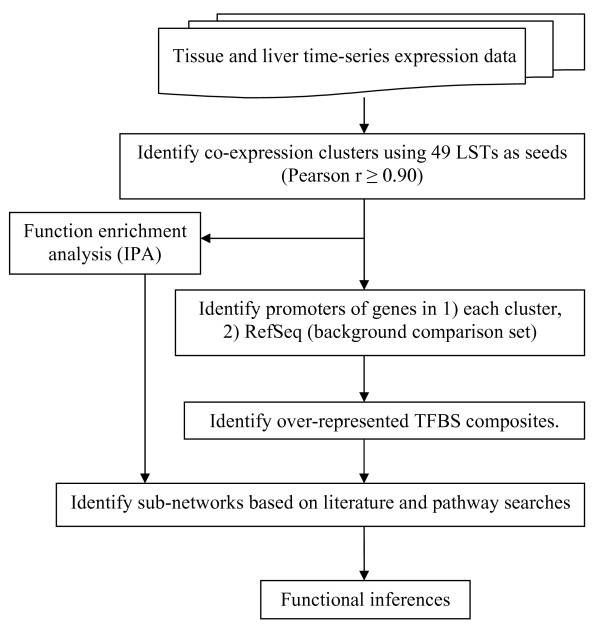
**Schema for inferring functions of LSGs**.

**Figure 2:**
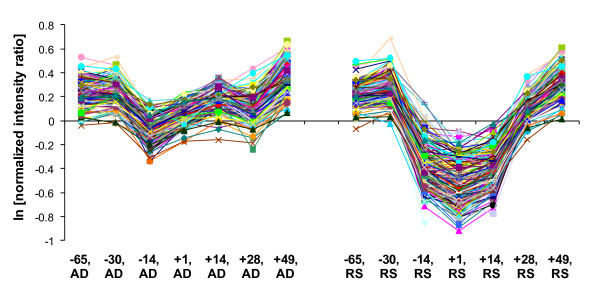
**Co-expression of LSTs and other genes in LIVR cluster**. The cluster of 212 genes (LIVR) shows a significant (P < 0.0001) difference in expression (~1.8-fold) between *ad-libitum *(AD; left panel) and energy-restricted (RS; right panel) diets at +1 and +14 days post-partum.

**Figure 3:**
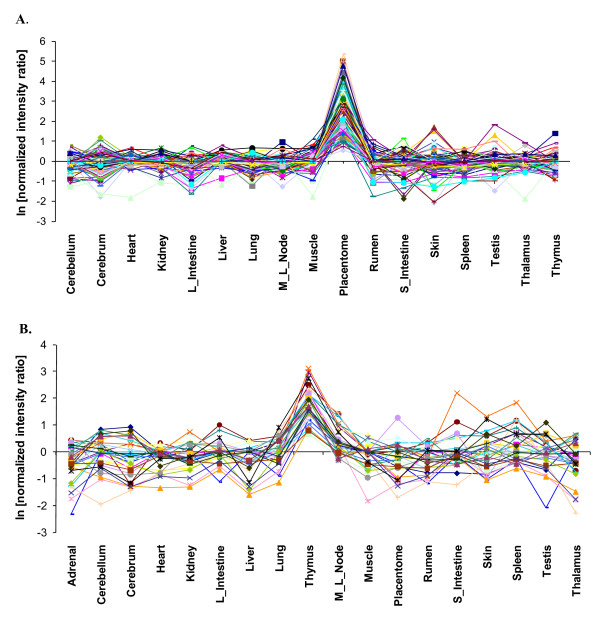
**Co-expression of genes in PLAC and THYM clusters**. The average pairwise Pearson correlation (r) within each cluster was r ≥ 0.75. The correlation between any one of the LSTs and any known gene was r ≥ 0.90: **A) **Co-expression of LSTs 22JE, 34FL, and 104JE with 113 other genes using expression data from 18 cattle tissues. A cluster of 116 genes (PLAC) shows preferential expression in placentome, with each gene having ≥ 2-fold higher expression in placentome as compared to any other tissue: **B) **Co-expression of LSTs 383NG and 21PW with 30 other genes using expression data from 18 cattle tissues. A cluster of 32 genes (THYM) shows preferential expression in thymus with each gene having ≥ 2 fold higher expression in thymus as compared to any other tissue. L_Intestine, large intestine; M_L_Node, mesenteric lymph node; S_Intestine, small intestine.

A strategy for inferring functions of LSTs (Figure 1) was applied to 63 previously identified LSTs [2] (see Methods). Two microarray expression datasets, consisting of profiles of ~7,000 cattle genes [21] and including these LSTs, were used for generating co-expression clusters. From the dataset consisting of profiles of total RNA from 18 cattle tissues, 49 LSTs and 6,178 known genes were selected for further analysis after applying filtering conditions (see Methods). Using the LSTs as seeds, two clusters were identified that showed preferential expression in a specific tissue with at least two-fold higher expression compared to any other tissue (Figures 2 and 3).

From the liver time-series dataset [22], 28 of the 49 LSTs that had tissue profiles and 4,711 known expressed genes were selected for clustering after data filtering (see Methods). Two large clusters were identified with average pairwise Pearson correlation r ≥ 0.75, and r ≥ 0.90 between any LST and transcripts encoded by known genes. The identity of the genes in these clusters overlapped, and instead of merging the clusters by lowering the correlation threshold, we selected the largest cluster containing four LSTs and 208 known transcripts (LIVR) for further analysis. These transcripts were co-expressed at seven time-points and two diets (Figure 2). Apart from liver, the genes in this cluster were expressed at higher levels in adrenal gland, cerebrum, and placentome (Additional file 1).

**Figure 4:**
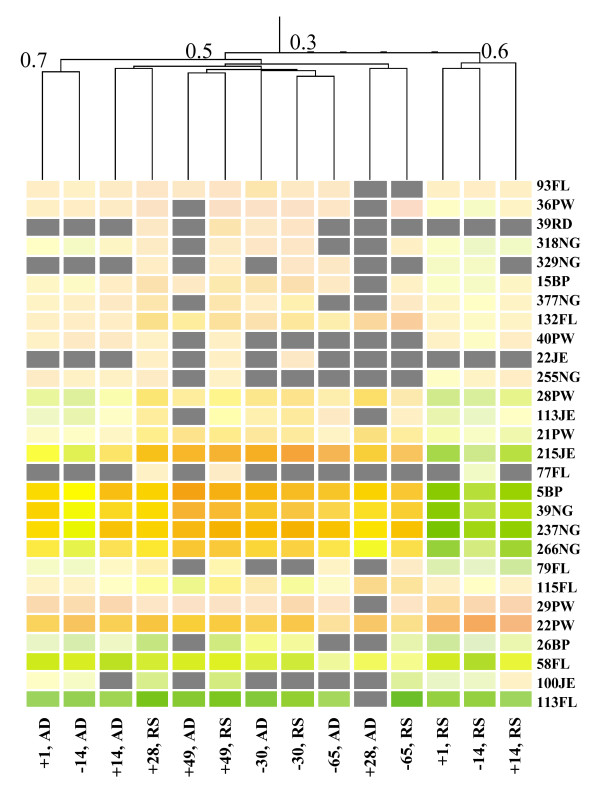
**Clustering of 28 LSTs significantly expressed (P < 0.05) at one or more time-point(s) and by diet**. Gray cells indicate that the gene is either not expressed at that time-point/diet, had missing data, or did not meet the filtering criteria (see Methods). The numbers at the intersection of branches indicate the branch-correlation. Green cells indicate under-expression, orange/red/pink cells indicate over-expression, and yellow cells indicate no change in expression compared to the reference sample.

In order to identify plane of nutrition (diet) and time-dependent relationships from the liver time-series data [22], the 28 LSTs that passed filtering of the liver expression data were hierarchically clustered (Figure 4). The four LSTs in the LIVR cluster (5BP, 39NG, 237NG, 266NG) were down-regulated at -14, +1, and +14 days relative to parturition in animals fed a restricted-energy diet pre-partum (Figure 4). Analysis of the entire LIVR cluster for diet and time-dependent relationships indicated that the genes were under-expressed by 1.8-fold at +1 and +14 days after parturition in liver of animals on a lower (restricted) as compared to higher (ad libitum) plane of nutrition pre-partum (P < 0.0001). This suggests that the expression of the LIVR cluster during the peri-partum period is directly influenced by plane of nutrition pre-partum (Figure 2).

### LIVR cluster and functional inference for LSGs

The LIVR cluster contains 208 known genes and four LSGs, *237NG, 266NG, 39NG*, and *5BP *(Table 1; Figure 2). To determine the subsets of genes in the LIVR cluster that may be co-regulated, we predicted and analyzed TFBSs and their composites in upstream promoter regions [-100, 1000] relative to the TSS using cattle genome (BCM_HGSC Btau_3.1) [23] sequence. p53 and Oct-1 TFBSs were found to be significantly over-represented in the LIVR cluster. The Oct-1 site had a high frequency in the cluster and was predicted in 40 genes including the LSG *39NG*. Of the over-represented paired composite TFBSs, Srebp-1*Pax-8 was predicted in the upstream region of two LSGs, *237NG *and *266NG*, and known genes *CCDC12 *(coiled-coil domain protein 12), *MX1 *(interferon-inducible protein p78), *NGLY1 *(N-glycanase), *PLCE1 *(phospholipase C, epsilon 1), *TRIP10 *(*CDC42*-interacting protein 4), and *ZDHHC18 *(zinc finger, DHHC domain containing 18). A search of the composite regulatory signature discovery (CRSD) database [24] for TFs Srebp-1 together with Pax-8 identified glycerophospholipid metabolism, among others, as over-represented pathways (Table 2). The cluster was enriched for another paired composite, Sp1*Pax-8, which was predicted in the LSG *237NG *and five other known genes. This suggests that *237NG *is regulated by all three TFs, Sp1, Srebp-1 and Pax-8.

**Table 1:** Summary information for nine LSTs co-expressed with known genes.

LST	**GenBank acc**.	**Cluster**^**a**^	Btau_3.1	Length (LST)	Exon	**CDS**^**b**^	**Species- specifcity**^**d**^
22JE	EU998973	PLAC	chr7:65,670,509-65,672,078	1569	1	ncRNA	Bt, Ss
104JE	EU998975	PLAC	chr3:78,876,417-78,886,469	619	5	68	Bt, Ss, Oa
34FL	EU846101	PLAC	chr29:24532031-24538612	1571	2	100K^c^	Bt, Ss, Oa, Ch, Ec,
383NG	EU998980	THYM	chr8:31,549,088-31,549,951	864	1	61K^c^	Bt, Oa
21PW	EU998981	THYM	chr26:12,357,963-12,358,929	977	1	67	Bt
5BP	EU998982	LIVR	chr3:92,641,001-92,641,610	610	1	ncRNA	Bt
237NG	EU998978	LIVR	chr19:51,623,121-51,623,742	622	1	62	Bt
39NG	EU998977	LIVR	chr3:79,052,419-79,054,067	767	2	172K^c^	Bt
266NG	EU998979	LIVR	chr12:29,282,078-29,283,427	783	2	38K^c^	Bt

^a ^PLAC, placenta; THYM, thymus; LIVR, liver

^b ^A CDS, length of coding sequence in amino acids.

^c ^A Kozak consensus sequence is predicted at the beginning of the ORF.

^d ^Bt, *Bos taurus *(cattle); Ss, *Sus scrofa *(pig); Oa, *Ovis aries *(sheep); Ch, *Capra hircus *(goat); Ec, *Equus caballu*s (horse).

**Table 2:** Over-represented ordered TFBS pairs and unordered TFBS triplets in LIVR, PLAC and THYM co-expression clusters.

Cluster	^**a**^**TFBS singles and pairs**	P-value	**P-value**^**c**^	**Ref**.	**CRSD pathway (<10**^**-03**^**) and ** **PREMOD identifier**^**d**^
LIVR	Oct-1^b^	0.002	0.152	[37]	NA^e^
	p53	0.002	0.152	[36]	NA
	Sp1*Pax-8	0.001	0.049	[72]	Agrin in postsynaptic differentiation; glycerophospholipid metabolism
	SREBP-1*Pax-8^b^	0.002	0.049	[40]	glycerophospholipid metabolism; Agrin in postsynaptic differentiation
	ZF5*YY1	0.002	0.049	[73]	Wnt signaling pathway; antisense pathway
	Ebox*c-Ets-1(p54)	0.004	0.052	[74]	nicotinate and nicotinamide metabolism; signal transduction
	AP-2, ZF5, c-Ets -1(p54)^b^	0.027	0.026	[43]	adipocytokine signaling pathway; HIV-I Nef: negative effector of Fas and TNF
PLAC	STAT*Pax-2^b^	0.0009	0.10	[75,76]	glycerolipid metabolism (with STAT family); prion pathway; mod027529
	Tax/CREB*ETF	10^-05^	0.039	NA	EGFR-specific transcription factor (ETF) not found in CRSD
	Oct-1*GATA-4	0.0009	0.10	[77]	mod003360; mod065501; mod070287
	Tel-2*VDR	0.0005	0.10	NA	Phosphatidylinositol signaling system; mod100969
THYM	v-Myb^b^	10^-05^	0.069	[78]	NA
	KROX	0.004	0.224	[79]	NA
	Nkx2-5*CdxA^b^	0.0006	0.077	[54]	N-glycan biosynthesis; ribosome; mod004754
	MAF*HOXA7	0.0002	0.077	NA	phospholipase C-epsilon pathway

^a ^* indicates order in this composite; ',' within the TFBS composite indicates an unordered composite.

^b ^Indicates that this composite is also predicted in an LST.

^c ^FDR corrected P-value

^d ^mod#, PREMOD identifier representing a predicted TFBS module.

^e ^NA, not applicable.

An unordered triplet composite of TFBSs (AP-2, ZF5, c-Ets1) that was over-represented in the cluster, was predicted in LSG *237NG *and five other known genes (*ANKRD16, ARF5, TMEM14C, ARL4A, NSMCE4A*). The three TFs that bind to those sites were found to be active in the adipocytokine signaling pathway on the basis of a CRSD search [25]. Comparison of the motifs predicted *ab initio *using *ANN-Spec *[26] with known TFBSs identified Elf1- and Sp1-like sites as matches, indicating that Elf1 and Sp1 TFs regulate genes in the LIVR cluster (Additional file 2). As corroborating evidence, an Elf1 binding site was predicted in 13% of the genes in the LIVR cluster, including *266NG *and *5BP*, and an Sp1 site was predicted in 27% of the genes, which included the LSG *237NG*. Analysis of the LIVR cluster using Ingenuity Pathway Analysis (IPA) [27] showed it to be enriched for genes in the *glycerophospholipid metabolism pathway, DNA repair, cell death, organ development of epidermis and immune response *(Table 3).

**Table 3:** Ingenuity Pathway Analysis of gene clusters.

**LST cluster**^**a**^	**Significant **^**b **^**functions (F) and canonical pathways (C)**	Genes included in the function
LIVR (143/212)	glycerophospholipid metabolism (C)	*LYPLA1, PGS1, PLCE1, PLCL2*
	cancer (F)	*FABP5, GLRX, GRRP1, MET, MLH1, PLCE1, PLXNB2, ASB2, KCTD11, CDK3*
	repair of DNA (F)	*CDC5L, ERCC1, MLH1, NHEJ1, NTHL1, POLI, XRCC1*
	immune response of organism (F)	*CD48, GATA3, MX1*
	development of epidermis (F)	*ALDH3A2, FABP5, GJB5*
PLAC (64/116)	Wnt/beta-catenin signaling (C)	*CDH1, CSNK1G2, DKK1, TLE4*
	acute-phase response signaling(C)	*FOS, HMOX2, PTPN11, SOD2*
	tissue morphology--size (F)	*CDKN1C, DLX5, IGF2, STC1, PTGS2, FOS, CDH1*
	small molecule biochemistry- transport of amino acids and synthesis of prostaglandin (F)	*SLC7A3, STX1A, COMT, PTGS2, IGF2, FOS, IGFBP7, CYP4A22, BCAT, STC1, MAN2A1, PTPN11, TFPI, SOD2*
	embryonic development-- proliferation and formation of embryonic tissue (F)	*ESM1, MED28, PTGS2, CDH1, DKK1, FOS, HAND1*
	development of embryonic and trophoblast cells (F)	*CDKN1C, HAND1, IGF2, PTPN11*
	cell cycle--entry into cell stage (F)	*CDH1, CDKN1C, FOS, MAD2L1, PTPN11, SOD2*
	lipid Metabolism	*CYP4A22, IGF2, IGFBP7, PTGS2, STC1, PTPN11, COMT*
	cell adhesion (F)	*CASK, CD151, CDH1, IGFBP7, MAD2L1, MAN2A1, PTPN11, PVRL2, TFPI*
	transcription (F)	*CASK, CDH1, CDKN1C, DKK1, DLX5, FADD, FOS, GATAD2A, HAND1, IGF2, MED28, MSX1, PTPN11, RP13-122B23.3, SNAPC2, SOD2, SPEN, TARBP2, THOC4, TLE4, UBTF, ZNF281*
	cancer--cell death of tumor cell lines(F)	*CDH1, CDKN1C, DKK1, FADD, FOS, IGF2, IGFBP7, IHPK2, MAD2L1, MSX1, PTGS2, PTPN11, SOD2, UBTF*
THYM (18/32)	cellular growth and proliferation (F)	*BTG1, CDCA7, ELF1, HMGB1, NCOR2, PCNA, PTK2, TCF12, ZFP36L2, ASXL1*
	cell death (F)	*PCNA, TRAP1, PLA2G7, BTG1, NCOR2, HMGB1, PTK2, TCF12, ZFP36L2*
	gene expression--transcription and transactivation (F)	*HMGB1, HMGB2, PCNA, ELF1, ASXL1, NCOR2, ZBTB7A, BTG1, PTK2, TCF12, NXF1*
	immune and lymphatic system development and function (F)	*HMGB1, TCF12, PTK2, CDCA7, NCOR2*

^a ^Numbers in parentheses indicate count of genes with IPA functions/cluster size; LIVR cluster, [5BP, 39NG, 237NG, 266NG] and 208 other genes; PLAC cluster, [104JE, 22JE, 34FL] and 113 other genes; THYM cluster, [383NG, 21PW] and 30 other genes.

^b ^All P-values were < 0.05

### PLAC cluster and functional inference for LSGs

The PLAC cluster was expressed preferentially in placentome and consisted of 116 genes, including three that are LSGs, *34FL, 22JE*, and *104JE *(Table 1; Figure 3A). On the basis of PSI-BLAST search [28] and multiple sequence alignments we have annotated one of the LSTs, 34FL [GenBank: NM_001105478], as an *SSLP-1 *(secreted seminal vesicle protein) homolog, which belongs to a class of secreted Ly6 domain containing proteins. The predicted protein product of 34FL, like the SSLP-1 glycoprotein in mouse [29], has 10 cysteines and contained the conserved C-terminal CCXXXXXCN motif, indicating that it is a member of the SSLP-1 secreted Ly-6 glycoprotein subfamily (Additional file 3). In addition, 34FL was predicted by PSORTII [30] to contain a signal peptide, and was localized to the extracellular region providing evidence that it is a secreted protein. Furthermore, the *34FL *gene was located on BTA29 in an orthologous region that is syntenic with mouse *SSLP-1 *on MMU9.

The PLAC cluster was not found to be enriched for any single TFBS or TFBS triplets. However, we identified four TFBS composite pairs in the cluster (Table 2). The pair, STAT*Pax-2 (signal transducer and activator of transcription; paired homeobox 2), was predicted in the LSG *34FL*, *PAG2 *(Pregnancy associated glycoprotein), and *PTGS2 *(COX2, prostaglandin-endoperoxide synthase 2). The motifs predicted *ab initio *by *ANN-Spec *in the cluster had significant matches to NF-κB (nuclear factor kappa B), MAZ (Myc-associated zinc finger), and Sp1 TFBSs. All three sites were predicted in the cluster at varying frequency, although none were predicted in an LSG (Additional file 2). The cluster was found to be enriched for Wnt/β-catenin signaling and acute phase response (APR) signaling pathways. Other enriched IPA functions in the PLAC cluster were *transport of amino acids and synthesis of prostaglandins, adhesion, development of trophoblast cells, and lipid metabolism*.

### THYM cluster and functional inference for LSGs

A thymus-specific cluster (THYM) was identified, consisting of 32 genes, including two LSGs *383NG *and *21PW*. Both of these are single-exon transcripts (Figure 3B) and have multiple ESTs from different libraries as evidence of transcription. *383NG *is a paralog that has been duplicated in two other locations on the same chromosome [2]. The THYM cluster was found to be enriched for v-Myb (myeloblastosis viral oncogene homolog) and KROX (also EGR, early growth response gene) TFBSs (Table 2). Three TFBS composite pairs were over-represented in the THYM cluster, of which one, Nkx2-5*CdxA, was predicted in the LSG *21PW *and *ASXL1 *(Additional sex comb-like 1). An *ab initio *predicted motif matched the IRF (Interferon regulatory factor) TFBS. IRF-1 was identified in 21% of the genes in the cluster, including the LSGs *383NG *and *21PW *(Additional file 2). An analysis of the THYM cluster using IPA showed enrichment for genes involved in *apoptosis, immune and lymphatic system development, transcription and trans-activation, and cell proliferation *(Table 3).

### Gene interaction network for the LIVR cluster

**Figure 5:**
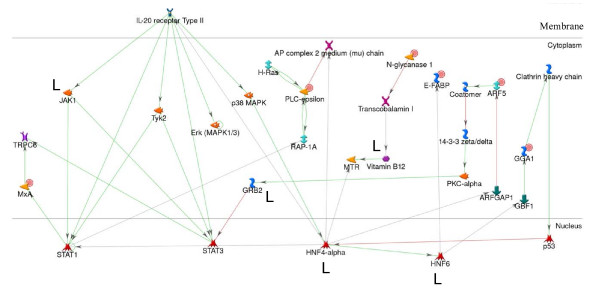
**Interaction network for co-expressed genes in liver and the LIVR cluster showing roles in glycerophospholipid metabolism, protein transport, and signaling**. LIVR cluster genes were analyzed using GeneGo MetaCore [32] and its human-specific interaction database. A sub-network was built starting with *PLCE1*, *NGLY1*, *MX1*, *TRIP10 *and *ARF5*, which are genes that are predicted to be co-regulated with *237NG *and *266NG*. Hub genes (*IL22RA1*, *GGA1*) predicted by WGCNA were then added to this network. Only interactions known to occur in liver tissue are shown as determined using a liver tissue trace in GeneGO. Majority of these are common to placenta tissue as determined using a placenta tissue trace. Those interactions that are specific to liver only are marked with the letter L. Genes that are expressed as part of the LIVR cluster are indicated with a red circle. A legend explaining the symbols is provided at http://portal.genego.com/legends/legend_6.png.

We then used weighted gene co-expression network analysis (WGCNA) [31] to identify sub-networks in the LIVR cluster. Only one module consisting of all the 212 genes was identified indicating the integrity of the cluster. On the basis of gene connectivity measurements, the top five hub genes (*CNGB3*, *GGA1, FABP5, IL22RA1, 237NG*) with the highest connectivity were identified in the LIVR cluster, and included the LSG *237NG*. The hub genes *CNGB3 *(cyclic nucleotide gated channel beta 3) and *GGA1 *(golgi associated, gamma adaptin ear containing, ARF binding protein 1) are known to play roles in ion and intracellular protein transport. The hub genes *IL22RA1 *(interleukin 22 receptor, alpha 1) functions in cell signaling and *FABP5 *(fatty acid binding protein 5, epidermal) in FA metabolism and signaling, suggesting that in addition to protein transport, these are dominant processes represented in the LIVR cluster. Using gene interactions from *GeneGO MetaCore *[32], which is modeled on known pathways in humans, a network was inferred for a subset of genes that are co-expressed with *237NG *and *266NG*. In addition, hub genes were added to build the network (Figure 5).

## Discussion

Functional elucidation of a novel gene is a challenging task. We have used an informatics-based strategy (Figure 1) to infer functions of a set of LSGs first found expressed in a cattle term-placenta cDNA library [2]. This was accomplished by generating co-expression clusters (Figures 2 and 3) using LSTs as seeds to cluster other genes from two microarray datasets consisting of transcript profiles from 18 cattle-tissues and liver of animals fed two different diets at several peripartal time-points. We then identified over-represented TFBSs and their composites in the promoters of co-expressed genes, and searched existing databases and the literature, for pathways and functions in which these TFs may play a role in a specific tissue or developmental stage. Yu and co-authors found that genes targeted by the same TF tend to be co-expressed, with the degree of co-expression increasing if genes share more than one TF [33]. This provides significant validation of our approach, and gives us confidence in the sub-networks of co-regulated genes that were identified. We present below a synthesis of our results with the aim of supporting the inferred functions of LSGs in each cluster.

### Evidence supporting inferred functions for LSGs in the LIVR cluster

The LIVR cluster was found to be enriched for genes in the glycerophospholipid metabolism pathway, DNA repair, transport, cell death, organ development of epidermis, and immune response functions (Table 3). These pathways and functions are also characteristic of term placenta [34], which was the source tissue used to create the cDNA library from which the LSTs were identified. In support of the correlated pathways and functions of genes in liver and placenta we also found that the LIVR cluster genes are expressed in placentome (Additional file 1). Glycerophospholipid metabolism plays a significant role in the onset of labor in humans [35], and apoptosis and immunological processes are known to represent important cellular functions in term-placenta [34]. The overlapping functions likely represent common subpopulations of cells in liver and placenta, such as macrophages and lymphocytes.

Genes in the LIVR cluster were enriched for p53 and Oct-1 TFBSs. p53 exerts a variety of regulatory effects following DNA damage [36]. An Oct-1 TFBS has been predicted in the *39NG *promoter along with a PPARγ site. PPARγ works in concert with Oct-1 to mediate transcriptional activation of *GADD45 *(growth arrest and DNA damage-inducible gene 45) [37]. The presence of both Oct-1 and PPARγ sites on the 39NG promoter suggests a role for the encoded protein in DNA repair processes in response to DNA damage. In addition, the protein is predicted by *PSORTII *[30] to be a nuclear protein, which supports such a role. A paired TFBS composite, Srebp-1*Pax-8, was significantly over-represented in the LIVR cluster, and was predicted in two LSGs, *237NG *and *266NG*. It was also predicted in *PLCE1, NGLY1*, and *TRIP10*, which have known roles in fatty acid (FA) metabolism, turnover of glycoproteins, and lipid binding, respectively (see Additional file 4 for protein functions). Srebp-1 is known to regulate genes involved in the biosynthesis of fatty acids, triglycerides and phospholipids in liver and adipocytes [38], and has been shown to play a role in glycerophospholipid metabolism [39] suggesting that *237NG *and *266NG *are also involved in these processes. Smith and coauthors reviewed evidence that show Pax-8 works together with Srebp-1 to target *PPARγ *(peroxisome proliferator-activated receptor gamma) in adipocytes and liver [40]. Some of these LIVR genes were shown to form sub-networks that participate in glycerophospholipid metabolism, protein transport and signaling pathways in liver (Figure 5). The LSG, *237NG*, is inferred to play a role in glycerophospholipid metabolism and cytokine signaling, and is one of the hub genes.

The LIVR cluster was enriched for an unordered triplet TFBS composite, AP-2, ZF5, c-Ets1 (p54), which included the LSG *237NG*, *ANKRD16, ARF5, TMEM14C, ARL4A*, and *NSMCE4A*. The transcription factor AP-2 (TFAP2A) is correlated with expression of cytokine-induced serum amyloid A1 gene (SAA1) in cattle liver [41] and is known to be a repressor for SAA1 [42]. SAA1 plays a role in the immune system, supporting a role for 237NG in cytokine-related immune processes. AP-2 and ZF5 binding sites have been predicted together in liver by Smith et al [43]. A sub-network of genes including *ARF5, ARFGAP1 *and the hub gene *GGA1*, which play roles in protein trafficking and localization within the golgi apparatus, was shown previously to be linked with processes in signaling and glycerophospholipid metabolism pathways in liver (Figure 5). A motif predicted *ab initio *by *ANN-Spec *shows a significant similarity to the TFBS for Elf1, which is predicted in 13% of the LIVR gene promoters, including the promoter regions of *266NG *and *5BP *(Additional file 2). The Elf1 TF plays a role in early liver development of mouse embryos [44], thus suggesting a similar role for these two LSGs (Table 4).

**Table 4:** Inferred biological functions of LSTs.

Cluster	LSTs	Function Inference
LIVR	5BP (ncRNA) 237NG 39NG 266NG	Involved in glycerophospholipid/fatty acid metabolism, cell signaling and protein trafficking in epithelial cells. 39NG possibly plays a role in DNA repair processes in response to DNA damage. Responsive to differences in pre-partum plane of nutrition at time-points +1, +14 after onset of lactation (Figures 2 and 4).
PLAC	104JE 22JE (ncRNA) 34FL	Preferential expression in placentome; involved in immune response, acute phase and inflammatory processes. 34FL is a pre-term and term placentome-specific SSLP-1 glycoprotein, possibly involved with PAG2 and PTGS2 in the final events before parturition at the feto-maternal interface.
THYM	383NG 21PW	Preferentially expressed in thymus and may play a role in immune system development and cell-proliferation. 21PW may play a role in gene activation in fetal thymus development.

Expression of the LIVR genes was found to be affected by pre-partum diet. They were down-regulated by restricted feeding at +1 and +14 days postpartum suggesting that the predicted functions (e.g., apoptosis, glycerophospholipid metabolism, DNA repair mechanisms, and cell signaling) are down-regulated during the early post-partum period when the animals are fed restricted diets that do not meet 100% of the estimated energy requirements during the non-lactating period. This management strategy is more successful in preparing the animal to the onset of parturition and lactation, and leads to lower incidence of metabolic disease [22]. Therefore, animals on a higher plane of nutrition (i.e. consuming diets to meet or exceed energy requirements) show increased inflammatory responses, apoptosis, and DNA repair; a conclusion shared by Loor and coauthors [22]. Above, we suggested that glycerophospholipid metabolism is a common function in liver and near-term placenta in animals approaching labor and delivery. Metabolic processes in both tissues have been shown to be affected by diet in non-ruminants. For example, in pregnant mice the FA composition in the mother's diet influences the maternal liver and fetal placenta FA composition [45,46]. These findings suggest that the LIVR genes, many of which are involved in FA-linked functions, protein transport and cell signaling, play similar diet-responsive roles in both liver and placenta of pregnant animals (Figure 5), given that nearly all (99%) of the LIVR genes, including the LSGs, are also expressed in the placenta (Additional file 1).

### Evidence supporting inferred functions of LSGs in the PLAC cluster

The PLAC cluster genes were found to be preferentially up-regulated in placentome and enriched for specific processes in the placenta; e.g. transport of amino acids and synthesis of prostaglandins, trophoblast cell adhesion, lipid metabolism, transcription, and cell proliferation (Table 3). The cluster is also enriched for acute phase response (APR) genes, which function to restore homeostasis. These APR gene products are a variety of serum proteins synthesized in increased amounts in response to trauma and infection. Given that labor and delivery result in oxidative and immunological stresses, with APR and apoptotic responses in placental tissue [47], APR enrichment provides a snap-shot of these processes in near-term placenta. The cluster is also enriched for Wnt/β-catenin signaling, which has been shown to play a central role in coordinating uterus-embryo interactions required for implantation in mouse [48].

The composite TFBS pair, STAT*Pax-2, was over-represented in three co-expressed genes; *34FL*, *PAG2*, and *PTGS2*. The PWM for the predicted STAT binding site is common to a range of STAT proteins that are involved in the development and function of the immune system and play a role in maintaining immune tolerance and tumor surveillance. PTGS2 is a biosynthetic isoenzyme that was shown in pregnant cows and guinea-pigs to be involved in intrauterine prostaglandin (PG) synthesis, which is crucial for the initiation of parturition [49,50]. *PTGS2 *was found to be 20-fold greater in cattle term placentomes (delivery at 260 days or later) compared with preterm placentomes (delivery between day 174 and day 260 of gestation) further supporting its role in parturition [34]. Given that our data show that *34FL *(a predicted SSLP-1 glycoprotein), *PAG2*, and *PTGS2 *are highly co-expressed and predicted to be regulated by STAT TFs, we suggest that 34FL also plays a role in pregnancy and/or parturition.

The *ANN-Spec *motifs predicted *ab initio *in the PLAC cluster have significant matches to TFBSs for NF-κB (nuclear factor kappa B), MAZ (Myc-associated zinc finger), and Sp1 (Additional file 2). NF-κB is known to initiate transcription for a variety of genes that are involved in immune response, acute phase and inflammatory processes [51]. It has been located in human fetal membranes and decidua at term and pre-term delivery [52]. The physiological expression of *COX-2 (PTGS2*) in rat trophoblast involves a sustained activation of *NF-κB*, and its inhibition abrogates the inducibility of *PTGS2 *[48]. This result functionally links *NF-κB *and *PTGS2 *with the other co-expressed genes in the PLAC cluster, suggesting a complex role for glycoproteins including 34FL in initiating and orchestrating the cell biology at the feto-maternal interface before parturition (Table 4).

### Evidence supporting functional inference for LSGs in the THYM cluster

The thymus is an immune system organ that is of central importance to the maturation of T lymphocytes. Genes in the THYM cluster are enriched for the related functions *immune system and lymphatic system development*, *cell death*, and *cellular growth and proliferation *(Table 3). The v-Myb TFBS was over-represented in the THYM cluster and predicted in LSG *383NG*. The *v-Myb *oncogene product causes late onset T cell lymphomas when expressed in the T cell lineage of transgenic mice [53], thus suggesting a role for this LSG *383NG *in cell-proliferation. The TFBS composite pair Nkx2-5*CdxA was over-represented in promoters of *21PW *and *ASXL1 *(*additional sex comb-like 1*). CdxA and Nkx2 have been shown to be markers for endoderm germ layer patterning during gastrulation, a process necessary for formation of the thymus [54]. The *AsxL1 *gene in *Drosophila *is required to maintain homeotic gene activation and silencing, and its homologs have been identified in mouse and found to be expressed in thymus [55]. The roles played by the TFs CdxA and Nkx2 in endoderm germ layer patterning, and that of ASXL1 in homeotic gene activation and silencing support a role for the LSG *21PW *in thymus development. Furthermore, the IRF-1 TFBS, which regulates *IL-15 *gene expression and influences the development of T-cells and natural killer cells in the thymus [56], is predicted in LSGs *383NG *and *21PW *(Additional file 2). Taken together, these findings implicate *383NG *and *21PW *in immune system development and cell-proliferation (Table 4).

## Conclusions

We selected the placenta as a model system to identify and functionally characterize novel LSGs because of its unique characteristics as a rapidly evolving physiological system in mammals. As we and others have shown, the placenta is a rich source of expressed LSGs and rapidly diverging genes [2,3,57-60]. Such genes are candidates for adaptive placental functions acquired by the ruminant lineage. We used a combination of cluster analysis, promoter analysis, WCGNA, and gene annotation to predict the functions of nine previously uncharacterized LSGs (Table 4) from a starting set of 49 (18%). The stringent analysis criteria produced unique and highly correlated gene expression clusters among 18 different tissues and across seven time-points and two diets in liver (Figures 2 and 3). The three clusters analyzed contained nine LSTs, seven of which are encoded by presumptive novel protein encoding LSGs and two are presumptive ncRNAs [2]. Our results represent a major advance in characterizing the novel LSTs expressed in bovine placenta and have yielded predictions of functions that are consistent with their putative role in ruminant reproductive and immune physiology.

As additional animal genomes are sequenced and the numbers of novel genes with unknown functions increases, our approach establishes a valuable precedent for future studies. We show that it is possible to identify and characterize a significant fraction of lineage-specific genes bioinformatically, which may guide hypothesis-driven experiments to determine their biochemical and cellular functions. These may in turn yield new insights into the role of LSGs in speciation and adaptive evolution.

## Methods

### Source datasets

In a previous study, 91 novel transcripts were identified in a cattle placenta cDNA library. These LSTs were characterized on the basis of their genomic distribution and annotation in Btau_2.0 and expression patterns in 18 cattle tissues [2]. For the present work, the annotation was updated to Btau_3.1 (December 2007) [23]. Of the original 91 LSTs, 63 currently have no matches to non-Cetartiodactyl sequences in public databases [61]. The remaining 28 transcripts were not considered in this study as they were re-annotated as representing divergent homologs.

Two cDNA microarray expression datasets profiling ~7,000 cattle genes were used. The cDNAs used for the array were selected from a near-term cattle placenta cDNA library [21]. The first dataset (GEO GSE3029) was obtained by profiling total RNA from 18 cattle tissues [21]. For this dataset, transcripts were included in the analysis if the intensity was above the median signal intensity of negative control spots present on the array, and in addition, the minimum intensity was 250 units in at least one sample-point. The second dataset [GEO: GSE3331] was generated by temporal gene expression profiling of liver RNA during the peripartal period in Holstein cows fed with a moderate energy ad-libitum, or restricted diet in which the animals were fed to consume ca. 80% of their calculated energy requirements from -65 days until parturition [22]. The temporal data spanned -65 to +49 days relative to parturition for animals receiving each diet. Expression levels of the transcripts were analyzed further if the intensity was above the median signal intensity of negative control spots present on the array, the minimum raw intensity was 150 in at least one sample-point, and the relative expression compared to the control was statistically significant in at least one sample-point with a raw P-value (P) < 0.05. For both datasets, only those intensity spots that were flagged as 'present' were included in the analysis.

### Tissue expression profile clustering

Among the 63 LSTs, 49 were present in at least one of the 18 tissues with a raw intensity of 250 (Additional file 5). In addition to these 49 LSTs, expression levels of 6,178 transcripts passed this filter. The LSTs were clustered using Pearson correlation (r) threshold of 0.90. A representative was selected from each cluster and the un-clustered LSTs were self-represented. Genes on the array that co-expressed with each of these LSTs at r ≥ 0.90 were grouped into clusters that included all co-expressed LSTs. The cluster was adjusted to bring the average cluster r ≥ 0.75.

### Clustering of temporal liver gene expression profile

Of the 49 LSTs, 28 were present in at least one liver sample with a raw intensity of 150 and significantly expressed at P < 0.05 compared to a control mixture of tissues excluding liver. Expression of 4,711 unique genes passed these filter conditions. The temporal profiles of the LSTs were clustered hierarchically using gene-condition clustering as implemented in *GeneSpring *[62]. The liver gene expression profiles of representative LSTs were used as seeds to identify co-expressed genes from the 4,711 genes on the array using Pearson correlation (r) at a threshold r ≥ 0.90. As before, the clusters were adjusted to bring the average cluster r ≥ 0.75.

A mixed effects model using the *SAS *procedure Proc MIXED (SAS Institute, Cary, North Carolina, USA) [63] was used on the 212 unique genes in the liver cluster to determine expression differences between groups of animals on two diets (moderate energy ad-libitum and restricted) at different time points (-65, -30, -14, +1, +14, +28, +49 days). The LOG2-transformed ratios were analyzed for each gene using a mixed model that included the fixed effect of diet within time point. Statistically significant P-values for the models were adjusted for multiple comparisons using the Benjamini-Hochberg false discovery rate (FDR) correction [64].

### Functional annotation and assignment of genome coordinates of genes in clusters

Functions, gene symbols and genome coordinates were assigned to each clone accession on the array using RefSeq (Btau_3.1) and human protein annotations in UCSC genome browser tables [65]. Manual curation of the clusters involved removing identical genes and using the UCSC browser to check if each gene was annotated with the correct gene symbol and genome coordinates. This manual inspection was crucial for ensuring the transcription start site of genes and their promoter regions.

### Promoter extraction

Mammalian regulatory elements are concentrated near transcription start sites (TSS). For this reason, promoter analysis was concentrated on the proximal promoter region, -1000 to +100 bp relative to the TSS. Both unmasked and repeat-masked promoter sequences [-1000, +100] were extracted for gene clusters from Btau_3.1 using the UCSC Genome Table browser. To identify TFBSs that are over-represented in the gene clusters, we used promoters of unique cattle RefSeq genes as the background set. The coordinates for a non-redundant set of Btau_3.1 RefSeq genes were downloaded from UCSC Genome Table Browser, and the proximal promoters [-1000, +100] were extracted as described for the clusters.

### Identification of transcription factor binding site (TFBS)

Vertebrate-specific TFBSs were predicted by scanning the repeat-masked promoters using the *Match *program [66] with a core similarity threshold of 0.9 and a matrix similarity threshold of 0.85. The promoters in each cluster were searched against a predefined matrix profile in the TRANSFAC Professional 11.4 database [67]. This database contained a set of 214 high-quality, vertebrate-specific, non-redundant position weight matrices (PWMs) with minimized false positives ("vertebrate_non_redundant_minFP" with high-quality matrices selected). Only a single occurrence of a TFBS was counted in each promoter, and the predicted TFBS counts in each cluster were compared to those in the cattle RefSeq promoter set using Fisher's exact test (FET), which is based on a hypergeometric distribution. The computed P-values were adjusted for multiple comparisons using the Benjamini-Hochberg FDR correction [64].

### Identification of over-represented co-occurring TFBS combinations

Two TFBSs were defined as co-occurring if they were distinct, non-overlapping, and their PWMs had a core similarity threshold of 0.9 and matrix similarity threshold of 0.85 in the output from the *Match *program. The *Match *output lists the PWM matches in their positional order on the promoter. Both ordered (A-B ≠ B-A relative to the TSS) and unordered (A-B = B-A) TFBS pairs and triplets were predicted separately, and the orientation of the TFBSs was ignored. To prevent double-counting, only a single occurrence of a combination was counted per gene. For the unordered combinations, non-redundancy was ensured by collapsing each identified combination in its sorted order (A-B-A or A-C-B or B-A-C = A*B*C), and then counting only a unique occurrence of the unordered combination within a gene promoter. Unordered TFBS composites were denoted with a comma separating the sites. Composite ordered TFBSs were denoted with an asterisk between the sites indicating that they were predicted to be co-occurring in that order in the promoter relative to the TSS. TFBS pairs and triplets were predicted for three different minimum threshold distances of 20 bp, 50 bp, and 100 bp between the TFBSs to identify all adjacent non-overlapping TFBS combinations [16]. The maximum allowed inter-TFBS distance was set to 250 bp.

The counts of the ordered pair and triplet TFBSs were computed for each cluster of genes and compared to the counts of the respective pairs and triplets in the background RefSeq promoters using Fisher's exact test. A minimum cell count of five was necessary for comparisons. The computed P-values were adjusted for multiple comparisons using the FDR correction as before, and comparisons were deemed significant if the adjusted P-value was ≤ 0.1. The entire analysis was carried out for both repeat-masked and unmasked promoters and significant predictions in the repeat-masked promoters had to be predicted in the unmasked cluster promoters to be selected. This precautionary measure ensured that no predictions were within repeats. TFBS prediction results were manually checked for the presence of over-represented composites.

### *Ab initio *motif prediction and comparison to known TFBSs

*ANN-Spec *[26] was used for *ab initio *prediction of motifs that were common to an entire cluster. For each cluster of genes, the motif predictions were made on unmasked promoters, using the unmasked Btau_3.1 RefSeq promoters from which the cluster genes were subtracted as background. *ANN-Spec *was run iteratively by varying the predicted motif length from 6 to 16 bp and setting the run cycle (parameter m) to 100. The PWMs of predicted motifs were parsed from the *ANN-Spec *output. *Tomtom *[68] was used to compare the predicted PWMs with TRANSFAC v11.4 PWMs and comparisons with P < 0.01 were deemed significant. Logos depicting the frequency of each nucleotide at each position of a motif were generated for the *ANN-Spec*-predicted and corresponding matching Transfac PWMs using the *EnoLOGO *web server [69].

### Functional classification of clusters

Ingenuity Pathway Analysis (version 5.5) [27] was used to identify functional enrichment in the clusters, using the respective source gene sets (6,149 genes in tissue experiments, 4,711 in liver time-series experiments) as reference. The Ingenuity Pathway Knowledge Base (IPKB) was used as the source database for biological function and pathway assignment to genes. The significance threshold for function and pathway enrichment was P ≤ 0.05.

To identify known pathways in which the TFs were involved we queried the CRSD, which consists of miRNA, TF and gene expression regulatory signatures assigned to specific BioCarta and KEGG pathways using genome-wide enrichment analysis [24]. A Perl script was written that accepted a TFBS composite and parsed the dataset for the co-occurrence of the TFs in the composite in a common pathway at a P < 10^-03^. In addition, we used the Predicted Regulatory Module (PREMOD) database [70] to identify any known modules within our set of TFBS composites.

### Identification of genes with the highest connectivity using WGCNA

To identify sub-networks of co-expressed genes in the LIVR cluster and "hub genes" we used WGCNA (Weighted Gene Co-Expression Network) [71]. Expression ratios and log10 transformed P-values were used as input for the 212 genes in the LIVR cluster. WGCNA uses Pearson correlation to calculate an adjacency matrix using the power adjacency function defined as follows [71]:

where a_ij _is the adjacency between two genes i and j, x is the expression of a gene, and β is the power factor for a scale-free network. For the LIVR cluster, this power was 8 as determined by the scale-free network criterion provided by the authors [71]. Default parameters were used for module generation. Gene connectivity was determined, and the top five genes with the highest connectivity (hub genes) were identified using 1.2 as a cutoff for gene significance and intramodular connectivity (K/Kmax) cutoff of 0.95.

### Network inference using GeneGO

*GeneGO MetaCore *[32] was used to identify known interactions in the LIVR cluster of genes, modeled on the human interaction database included in GeneGO.

## List of Abbreviations used

LST: Lineage-specific transcript; LSG: Lineage-specific gene; ncRNA: noncoding RNA; TFBS: Transcription factor binding site; TF: Transcription factor; PWM: position weight matrix; LIVR: cluster of genes expressed in liver and showing effect of diet; PLAC: cluster of genes preferentially expressed in cattle placenta; THYM: cluster of genes preferentially expressed in cattle thymus; SKIN: cluster of genes preferentially expressed in cattle skin; ADRBRN: cluster of genes preferentially expressed in cattle adrenal gland, thalamus, and cerebellum; FET: Fisher's exact test; FDR: Benjamini-Hochberg false discovery rate.

## Authors' contributions

CGK and HAL conducted the research, analyzed the results, and wrote the manuscript, REE contributed to data analysis, and JJL provided the liver time-series data and participated in the analysis. All authors have read and approved the manuscript.

## Supplementary Material

Additional file 1 **Tissue expression profile of the LIVR cluster of genes**. Tissue expression profile of the LIVR cluster of genes includes two of the LSTs (237NG, 5BP) and 104/208 other genes in the cluster. L_Intestine, large intestine; M_L_Node, mesenteric lymph node; S_Intestine, small intestine. Click here for file

Additional file 2 **Comparison of ANN-Spec. *ab initio *predicted motif PWMs with known Transfac binding site PWMs**. This table displays the frequency logos of PWMs that were predicted using *ANN-Spec *and for known Transfac binding sites with which the predicted PWMs were significantly matched. Click here for file

Additional file 3 **Multiple alignment of 34FL with mouse SSLP-1**. Multiple alignment of 34FL with mouse SSLP-1 and other secreted Ly6 domain containing proteins having 10 conserved cysteine residues. Dashes in the alignment indicate gaps, and gray shaded areas indicate conserved cysteines. Click here for file

Additional file 4 **Functions of gene products**. Text file containing glossary of gene functions. Click here for file

Additional file 5 **LSTs used for clustering with tissue expression data**. Initial set of 49 LSTs used as seeds to cluster the tissue expression data. Click here for file
