# Supplementary figures and images for: Functional annotation of novel lineage-specific genes using co-expression and promoter analysis
Source: BMC Genomics. 2010 Mar 9;11:161. doi: 10.1186/1471-2164-11-161 (PMC2848242; doi:10.1186/1471-2164-11-161)

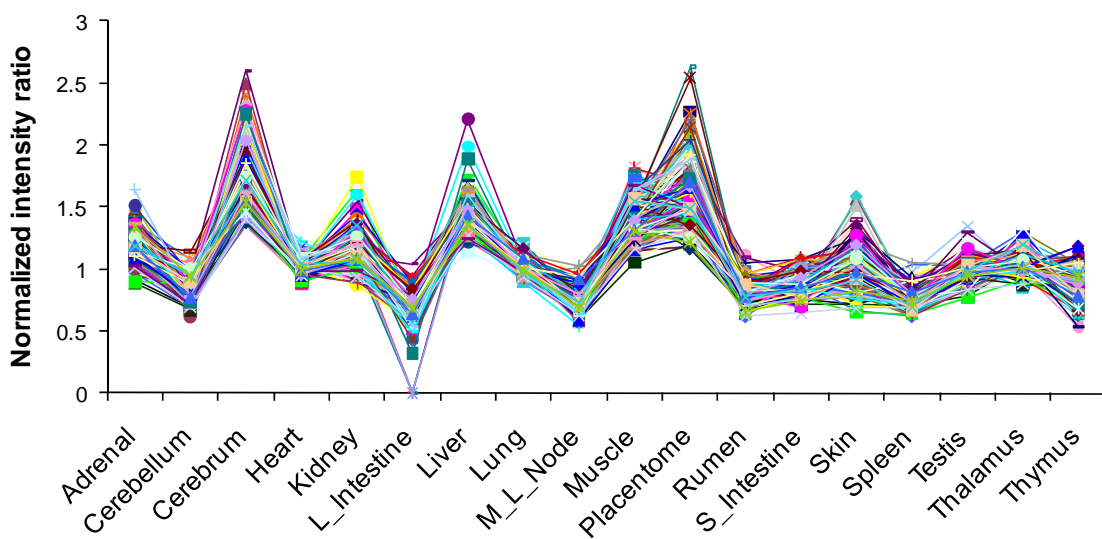

Supplement: Additional file 1 — Tissue expression profile of the LIVR cluster of genes. Tissue expression profile of the LIVR cluster of genes includes two of the LSTs (237NG, 5BP) and 104/208 other genes in the cluster. L_Intestine, large intestine; M_L_Node, mesenteric lymph node; S_Intestine, small intestine. [file 1471-2164-11-161-S1.PDF]
