# Supplementary material for: Functional annotation of novel lineage-specific genes using co-expression and promoter analysis
Source: BMC Genomics. 2010 Mar 9;11:161. doi: 10.1186/1471-2164-11-161 (PMC2848242; doi:10.1186/1471-2164-11-161)
Supplement: Additional file 2 — Comparison of ANN-Spec. ab initio predicted motif PWMs with known Transfac binding site PWMs. This table displays the frequency logos of PWMs that were predicted using ANN-Spec and for known Transfac binding sites with which the predicted PWMs were significantly matched. [file 1471-2164-11-161-S2.PDF]

Additional file 2. Comparison of ANN-Spec *ab initio* predicted motif PWMs with known Transfac binding site PWMs.

Cluster[a]	Predicted ANN-Spec motif in cluster	Transfac motif significantly matched by Tomtom[b]	Function[c]	TFBS % in cluster[d]	LSTs with TFBS[e]
L	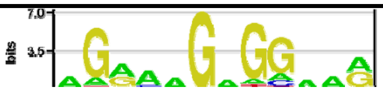	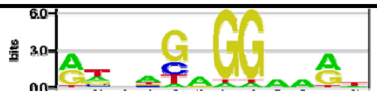	M00746 Elf1	13.7	2
L	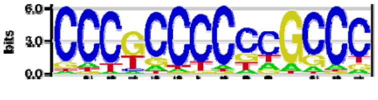	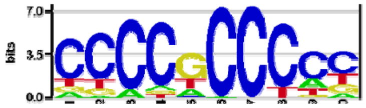	M00933 Sp1	27.5	1
P	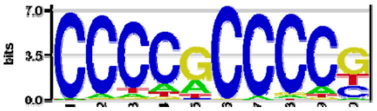	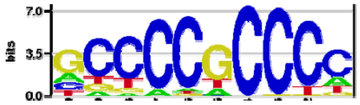	M00931 Sp1	19.0	0
P	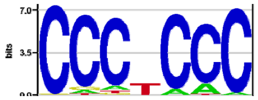	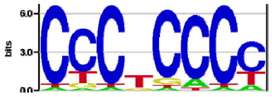	M00649 MAZ	27.0	0
P	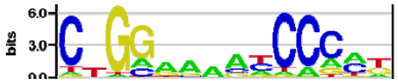	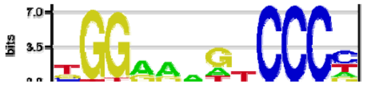	M00208 NF-kB	2.5	0
P	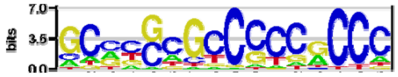	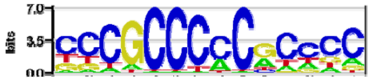	M00982 KROX	11.0	0
T	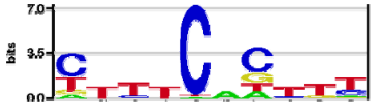	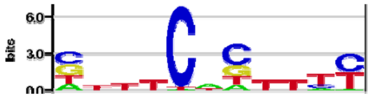	M00972 IRF	21.0	1
T	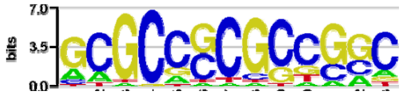	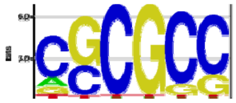	M00803 E2F	9.0	0
T	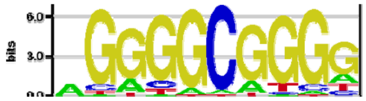	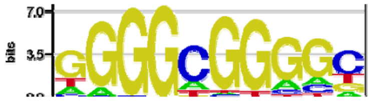	M00931 Sp1	28.0	0
T	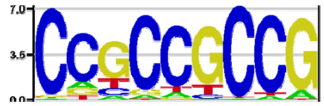	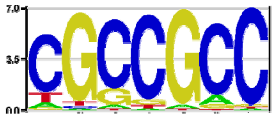	M01057 ERF2	31.0	0

[a] L, LIVR; P, PLAC, T, THYM.

[b] All P-values were ≤0.007

[c] Transfac TFBS matrix identifier and name.

[d] The entire Transfac database was used to search for TFBS matches. TFBS frequency was determined by taking the ratio of counts of the number of genes in which the TFBS was predicted (Core matrix score ≥0.95, PWM score ≥0.85) to the total cluster size.

[e] The number of LSTs in which the TFBS was predicted by Transfac.
