# Supplementary material for: Functional annotation of novel lineage-specific genes using co-expression and promoter analysis
Source: BMC Genomics. 2010 Mar 9;11:161. doi: 10.1186/1471-2164-11-161 (PMC2848242; doi:10.1186/1471-2164-11-161)
Supplement: Additional file 3 — Multiple alignment of 34FL with mouse SSLP-1. Multiple alignment of 34FL with mouse SSLP-1 and other secreted Ly6 domain containing proteins having 10 conserved cysteine residues. Dashes in the alignment indicate gaps, and gray shaded areas indicate conserved cysteines. [file 1471-2164-11-161-S3.PDF]

```
BTC1_34FL_Cattle      MAKCLLLLLLVVLSSLLGLPQALECFQCNRVNASGVCETGGSTCQTQGSQQCFLRRIFE- 59
SSLP-1_Mouse          MEKYLLLLLLGIFLR-VGFLQALTCVSCGRLNSSGICETAETSCEATNNRKCALRLLYK- 58
RUP-1_Rat             MGKPILLLPLGLSLL-MSSLLALQCFRCESLDSTGLCRVGRRICQTYPDEICAWVVVTTR 59
SLURP-1_Mouse         MTLRWAMWLLLLAAWSMGYGEAFRCYTCEQPTAINSCKN---IAQCKMEDTACKTVLETV 57
                      *     :  * :    :.   *: *  *    : . *.     .:   .  .    :

BTC1_34FL_Cattle      NGTLSYGH-----QGCSQLCIPMKLFNPSVIVEYKCCHDSPLCNKF-------- 100
SSLP-1_Mouse          DGKFQYGF-----QGCLGTCFNYTKTNNNMVKEHKCCDHQNLCNKP-------- 99
RUP-1_Rat             DGKFVYGN-----QSCA-ECIGTTVEHGSLIISTNCCSATPFCNMVHP------ 101
SLURP-1_Mouse         EAAFPFNHSPMVTRSCSSSCLATDPDGIGVAHPVFCCFR-DLCNSGFPGFVAGL 110
                      :. : :.      :.*   *:       .:     **    :**
```
