# Supplementary material for: Functional annotation of novel lineage-specific genes using co-expression and promoter analysis
Source: BMC Genomics. 2010 Mar 9;11:161. doi: 10.1186/1471-2164-11-161 (PMC2848242; doi:10.1186/1471-2164-11-161)
Supplement: Additional file 5 — LSTs used for clustering with tissue expression data. Initial set of 49 LSTs used as seeds to cluster the tissue expression data. [file 1471-2164-11-161-S5.PDF]

Additional file 5. LSTs used for clustering with tissue expression data

LST	GenBank acc.	Cluster	Tissue preference	Btau_3.1	Length (LST)	Exons	CDS[a]	Species-specificity
100JE	AW463214	none		chr7:6,465,501-6,467,878	2381	1	92K[b]	cattle, pig
104JE	EU998975	PLAC	Placentome	chr3:78876417-78886529	619	5	68	cattle, sheep, pig
113FL	EU848421	none	Thalamus	chr2:114,344,004-114,347,222	1124	6	69K[b]	cattle, sheep, pig
113JE	AW464579	none		chr3:29,804,733-29,806,315	886	2	ncRNA	cattle, pig
115FL	AW461454	none	Thymus	chr3:97,692,870-97,693,860	962	1	36	cattle, pig
132FL	AW463728	none	Thymus	chr21:24,822,025-24,824,160	2174	1	ncRNA	cattle,sheep,pig, chevrotain
144JE	BF044206	none		chr16:47,923,071-47,923,902	832	1	150K[b]	cattle
146JE	EU998976	none	Muscle, Skin	chr21:59545652-59554476	3623	5	ncRNA	cattle, pig
149NG	AY563726	none	Skin, Testis	chr17:69,359,843-69,360,508	690	1	35	cattle
14RD	EU781031	none	Adrenal, Cerebellum, Thalamus	chr18:62460314-62469206	954	7	235K[b]	cattle, sheep, pig
15BP	AY563717	none		chrUn.003.1478:22,936-27,708	644	3	ncRNA	cattle, sheep
15FL	AW465001	none		chr10:22,164,477-22,165,390	630	1	ncRNA	cattle
203JE	FJ357702	none	Heart	chr14:76,286,963-76,292,032	773	3	129K[b]	cattle
215JE	BF044986	none		chr1:146,051,902-146,052,941	1038	1	109K[b]	cattle, sheep
21PW	EU998981	THYM	Muscle, Thymus	chr26:12,357,963-12,358,929	977	1	67	cattle
22JE	EU998973	PLAC	Placentome	chr7:65,670,509-65,672,078	1569	1	ncRNA	cattle, pig
22PW	AY563868	none		chr13:80213223-80214742	555	2	ncRNA	cattle
237NG	EU998978	LIVR	Cerebrum	chr19:51623121-51623742	454	1	62	cattle
255NG	AY563763	none	Muscle	chrUn.003.1477:65,910-66,549	651	1	64	cattle,sheep, bottlenose dolphin
266NG	EU998979	LIVR		chr12:29282078-29283427	783	2	38K[b]	cattle
26BP	FJ357710	none	Rumen	chr19:55,989,649-55,994,111	946	6	46K[b]	cattle, sheep, pig
286NG	AY563780	none	Placentome	chr14:943,986-944,892	918	1	184K[b]	cattle,sheep,pig, goat
28PW	BF045305	none		chr19:50,892,442-50,892,944	1058	1	ncRNA	cattle
29PW	AY563874	none		chr7:19,353,956-19,355,078	604	2	80	cattle
314NG	FJ357704	none		chr3:114,615,299-114,619,844	762	2	84	cattle

318NG	AY563795	none		chr4:66,243,060-66,243,599	540	1	112	cattle
329NG	FJ357705	none		chr25:38,912,025-38,912,740	645	2	118K[b]	cattle
34FL	EU846101	PLAC	Placentome	chr29:24532021-24538613	1571	2	100K[b]	cattle,sheep,pig, goat, horse
352NG	AY563816	none		chrUn.003.1243:67,055-67,688	642	1	ncRNA	cattle
36PW	AY563880	none		chr13:36628830-36814160	694	5	98K[b]	cattle
377NG	AY563828	none	Placentome	chr8:86,019,929-86,037,325	908	2	ncRNA	cattle, sheep, pig
381NG	AY563830	none		chr23:46,606,505-46,607,047	542	1	ncRNA	cattle
383NG	EU998980	THYM	M.L.node,Muscle, Spleen, Thymus	chr8:31,549,088-31,549,951	777	1	61K[b]	cattle, sheep
39NG	EU998977	LIVR		chr3:79052419-79054067	767	2	172K[b]	cattle
39RD	FJ357711	none		chr19:35422164-35427503	1323	2	111	cattle,pig, horseshoe bat
3BP	AW465740	none		chr21:59,637,842-59,642,957	1347	3	139	cattle
407NG	AY563846	none	Large_intestine, Muscle, Thymus	chr10:3,000,382-3,001,018	638	1	78	cattle
40PW	FJ357708	none	Placentome	chr13:76,227,508-76,238,253	984	4	113	cattle, sheep, pig
416NG	AY563852	none		chr10:70,674,712-70,677,830	845	2	71	cattle, sheep, pig
51JE	EU998974	none	Skin	chr29:43,431,489-43,432,613	958	2	130	cattle
57FL	AY563869	none	Thymus	chr25:38,449,472-38,450,431	888	2	ncRNA	cattle, sheep
58FL	AW463389	none		chr29:32610872-32616473	1684	2	64	cattle,sheep, bottlenose dolphin
5BP	EU998982	LIVR		chr3:92641001-92641610	610	1	ncRNA	cattle
77FL	AY563824	none	Thymus	chr24:58678709-58679796	1095	1	84	cattle
79FL	EU848420	none	Cerebrum	chr11:47578720-47583524	732	4	ncRNA	cattle
7BP	AY563744	none		chr7:73,958,459-73,959,198	759	1	ncRNA	cattle
8NG	FJ357703	none	Thalamus	chr19:54080737-54083305	703	4	71K[b]	cattle, goat, red deer
93FL	AY563890	none	Thymus	chr17:65447194-65448403	1269	1	147K[b]	cattle, pig
95FL	BF041555	none	Skin	chr23:8874056-8876545	2492	1	108	cattle, sheep

[a] CDS, length of coding sequence in amino acids;

[b] A Kozak consensus sequence is predicted at the beginning of the ORF
